# Expanding spectrum of “spitzoid” lesions: a small series of 4 cases with *MAP2K1* mutations

**DOI:** 10.1007/s00428-020-02940-3

**Published:** 2020-10-11

**Authors:** K. G. P. Kerckhoffs, T. Aallali, C. A. Ambarus, V. Sigurdsson, A. M. L. Jansen, W. A. M. Blokx

**Affiliations:** 1 Department of Pathology, Maastricht University Medical Center+, Maastricht, The Netherlands; 2 Symbiant Pathology Expert Center, Hoorn/Zaandam, The Netherlands; 3 Department of Pathology, Sint Antonius Hospital, Nieuwegein, The Netherlands; 4 Department of Dermatology, University Medical Center Utrecht, Utrecht, The Netherlands; 5 Department of Pathology, Division of Laboratories, Pharmacy and Biomedical Genetics, University Medical Center Utrecht, Utrecht, The Netherlands

**Keywords:** Spitz nevus, MELTUMP, Melanoma, MAP2K1

## Abstract

The molecular background of a significant proportion of spitzoid neoplasms is still unknown. Recently, activating mutations in *MAP2K1* have been described in a few spitzoid lesions, but not in benign Spitz nevi. We report four cases of melanocytic tumors with spitzoid features in which a *MAP2K1* mutation was detected. The lesions did not show a single distinct phenotype and ranged from benign to malignant. Two cases resembled desmoplastic Spitz nevi. Based on the combination of morphological, immunohistochemical, and molecular findings, one case was classified as benign, one as probably benign, possibly intermediate low-grade (MELTUMP—melanocytic tumor of unknown malignant potential), one case was classified as intermediate (MELTUMP), and one case was considered a superficial spreading melanoma with spitzoid features. Based on this, we conclude that *MAP2K1* mutations can indicate a spitzoid genetic signature and can be found in both benign and malignant spitzoid neoplasms.

**Electronic supplementary material:**

The online version of this article (10.1007/s00428-020-02940-3) contains supplementary material, which is available to authorized users.

## Background

During recent years, great progress has been made in analyzing the molecular background of melanocytic lesions, including spitzoid neoplasms. Spitzoid neoplasms are defined based on their histopathologic appearance. They show large epithelioid or spindled cells with specific architectural features. It was known that about 20% of the spitzoid tumors carry an *HRAS* mutation [1]. More recently, chromosomal rearrangement-induced fusions have been identified in 50 to 55% of spitzoid lesions involving the kinase genes *ROS1*, *ALK*, *BRAF*, *NTRK1*, *NTRK3*, *MET*, and *RE*T [2, 3]. While the genetics of a significant portion of spitzoid neoplasms has been identified, in a significant portion of cases, the driver event is still unknown. Quan et al. recently identified structural rearrangements in MAPK genes other than *BRAF* in eight spitzoid neoplasms [4]. Among these, they identified one case diagnosed as an atypical Spitz tumor (AST) with an activating in-frame deletion in *MAP2K1*. *MAP2K1* encodes MEK1, a serine-threonine and tyrosine kinase directly downstream of RAF. This kinase phosphorylates and activates ERK1/2 [5, 6]. Activating mutations in *MAP2K1* have been identified in melanoma [7, 8]. Raghavan et al. reported a *MAP2K1* mutation in a case they classified as spitzoid melanoma (malignant Spitz tumor, MST) [9]. Besides this, mutations in *MAP2K1* have been described in deep penetrating nevi (DPN) in combination with mutations in the beta-catenin pathway [10].

Herein, we report four cases of melanocytic tumors with spitzoid features in which a pathogenic *MAP2K1* mutation was detected.

## Material and methods

The four cases were identified during routine molecular work-up of spitzoid lesions. Three cases were sent in consultation to the Pathology Department of the University Medical Center in Utrecht (UMCU) (consultant pathologist WB). One case was initially seen in the Symbiant Pathology Expert Center in Alkmaar, one in the Meander Medical Center in Amersfoort, and one in the Sint Antonius Hospital in Nieuwegein. The fourth case was initially seen in the UMCU. Details about the immunohistochemical stains that were used can be found in Online Resource 1.

DNA was isolated from formalin-fixed paraffin-embedded (FFPE) specimens using the cobas® DNA Sample Preparation Kit (Roche) (cases 1–3) or the Maxwell RSC 48 instrument (Promega) (case 4) using the Maxwell® RSC FFPE Plus DNA Purification Kit (Promega), according to the manufacturer’s protocol. Subsequently, next-generation sequencing (NGS) and single nucleotide polymorphism (SNP) array analysis were performed (for details, see Online Resource 2).

### Case presentation

**Fig. 1:**
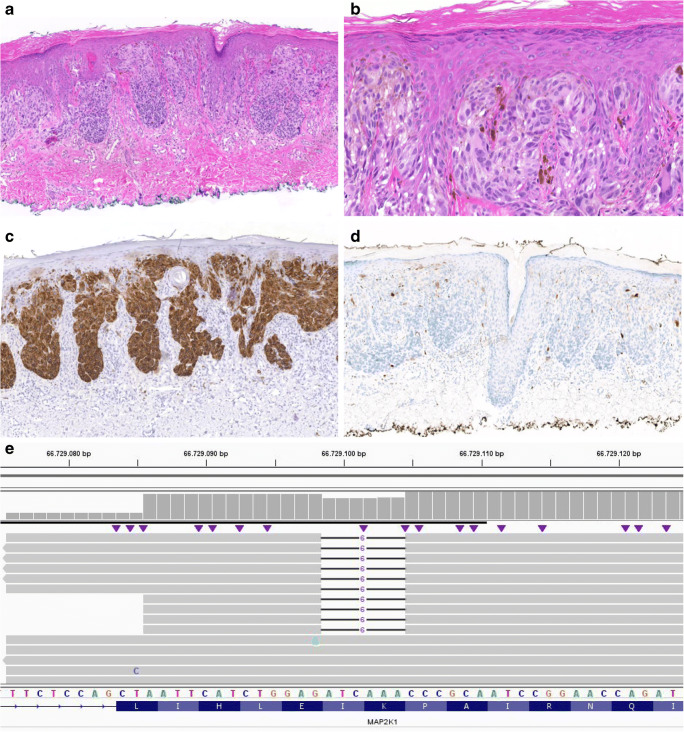
Microscopical and molecular findings case 2. **a** H&E, × 5: relatively symmetrical, compound melanocytic lesion extending into the superficial dermis with large nests at the junction. **b** H&E, × 40: epithelioid to spindle-shaped melanocytes with pale, eosinophilic cytoplasm. Variation in size and shape of the cells was encountered with large nuclei with prominent nucleoli and nuclear vacuoles. A few multinucleated cells were seen, **c** Melan A stain, × 5: diffusely positive. **d** p16 stain, × 5: negative in the majority of the lesion. **e** Integrative Genomics Viewer (IGV) visualization of the sequence data containing *MAP2K1* in-frame deletion c.307_312del (p.Ile103_Lys104del) in 24% of the reads (RefSeq NM_002755.3). The black lines indicate the location of the deletion

**Fig. 2:**
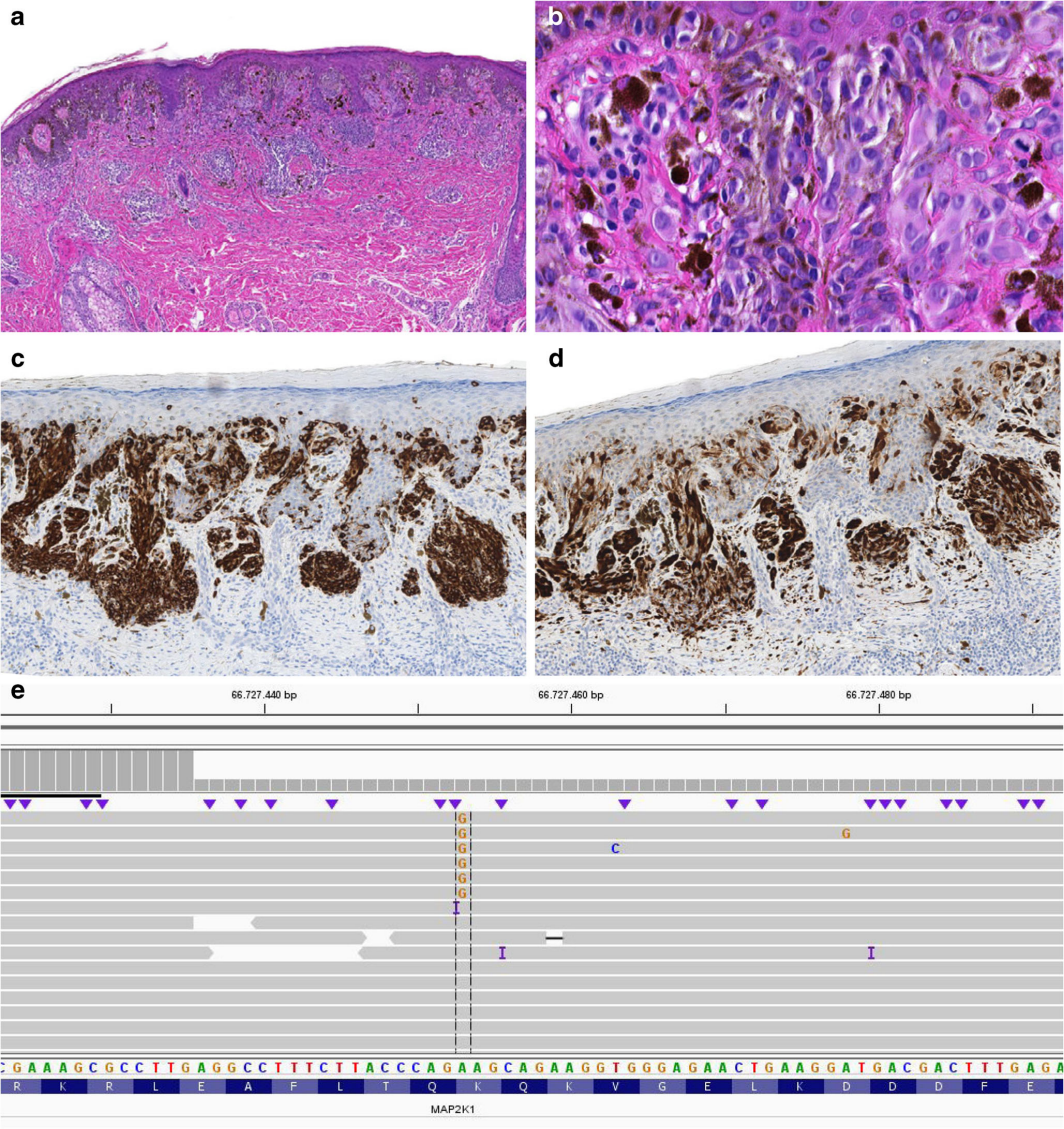
Microscopical and molecular findings case 3. **a** H&E, × 5: symmetrical, compound melanocytic lesion with nests and solitary melanocytes at the junction and a partly naevoid aspect of the dermal component. At the base melanocytes were spreading out between desmoplastic stroma. **b** H&E, × 40: epithelioid to slightly spindle-shaped melanocytes with pale, eosinophilic, partly pigmented cytoplasm. The cells had enlarged nuclei and presence of nucleoli was encountered. **c** Melan A stain, × 5: diffusely positive showing focal ascension. **d** p16 stain, × 5: positive in a checkerboard pattern**. e** Integrative Genomics Viewer (IGV) visualization of the sequence data containing *MAP2K1* mutation c.169A>G (p.Lys57Glu) in 7% of the reads (RefSeq NM_002755.3)

Detailed clinical information, microscopical and immunohistochemical findings, and the results of molecular testing of all four cases with a *MAP2K1* mutation can be found in Tables 1 and 2. None of the cases showed additional mutations. Furthermore, relevant images of cases 2 and 3 can be found in Figs. 1 and 2 (images of cases 1 and 4 are available in Online Resource 3). For cases 3 and 4, clinical pictures were available, see Online Resource 4.

**Table 1:** Clinical information and microscopical findings of all four cases

Case	Diagnosis	Age (y)	Sex	Relevant history	Site	Clinical appearance	Architecture	Epidermis	Cyto-morphology	Pigmentation	Mitotic figures	Nuclear atypia	Recurrence	Follow-up (months)
1	MELTUMP (perhaps AST)	17	F	No	Left lower arm	Elevated, round, erythematous papule. Diameter 8 mm. Had grown.	Symmetrical, dermal, slightly wedge-shaped. Desmoplastic stroma.	Hyperplastic	Epithelioid to spindle-shaped. Pale, eosinophilic cytoplasm.	No	0	Moderate to severe	No	4
2	SSM	16	M	No	Right wrist	Elevated, 2 × 2 mm, showing dark spot.	Symmetrical, compound, into superficial dermis. Large junctional nests. Few melanocytes in epidermis, no ascension.	Hyperplastic, hyperkeratosis	Epithelioid to spindle-shaped. Eosinophilic cytoplasm. Large nuclei, prominent nucleoli and vacuoles.	Slight	1 (junction)	Moderate	No	2
3	Variant Spitz nevus	48	F	No	Left upper leg	Clinical atypical nevus, black to brown, sharply demarcated, 5 × 4 mm (see clinical pictures).	Symmetrical, compound. Nests and solitary melanocytes at junction, focal ascension. In dermis partly smaller melanocytes with focally naevoid aspect. Desmoplastic stroma.	Hyperplastic, hyper- and parakeratosis	Epithelioid to spindle-shaped. Eosinophilic cytoplasm. Enlarged nuclei, nucleoli	Yes	1 (upper dermis)	Moderate	No	2
4	Probably Spitz nevus, at most low-grade MELTUMP (perhaps AST)	68	M	No	Right lower back	Nodule, variable pigmentation. Diameter 4 mm. Present for at least 4 years, unchanged (see clinical pictures). No clinical suspicion for malignancy.	Symmetrical, dermal, slightly wedge-shaped. Maturation towards deeper parts.	Normal	Spindle-shaped. Pale, eosinophilic cytoplasm.	No	0	No	No	7

*AST*, atypical Spitz tumor; *F*, female; *M*, male; *MELTUMP*, melanocytic tumor of unknown malignant potential; *SSM*, superficial spreading melanoma; *y*, years

**Table 2:** Immunohistochemical and molecular findings of all four cases

Case	Immunohistochemistry								Molecular testing			
	Melan A	HMB-45	ALK, ROS, NTRK	BRAF (V600E)	p16	p21	Proliferation fraction	BAP1	TCP	*MAP2K1* mutation - allele frequency	CNVs	
1	Positive	Weakly positive superficially	Negative	-	Few positive cells (checkerboard pattern)	Positive	Low (around 2%)	Positive	30–50%	c.306_311del (p.Ile103_Lys104del) - 13%	4^a^	Partial loss 2p Partial loss and partial CN-LOH 19p Chromothripsis 22q
2	Positive	Positive (slightly less staining deeper)	Negative	Negative	Negative (in majority of cells)	Positive (mainly at junction)	Low	Positive	30%	c.307_312del (p.Ile103_Lys104del) - 24%	6	Monosomy 9 Gain 6p Partial loss 6q Monosomy 10, 12, 19
3	Positive	Positive (partly maintained deeper)	Negative	-	Positive (checkerboard pattern)	Negative	-	-	10–20%	c.169A>G (p.Lys57Glu) - 7%	0	Probably not reliable due to low TCP
4	Positive	Negative	Negative	-	Focally positive	Positive	Very low	-	60%	c.306_311del (p.Ile103_Lys104del) - 28%	6	Trisomy chromosomes 5, 6, 11, 14, 15, and 20^b^

^a^Chromothripsis of chromosome 22 was counted as 1 CNV

^b^Due to poor quality of the SNP array, presence of other CNVs could not be excluded completely

*CN-LOH*, copy neutral loss of heterozygosity; *CNVs*, copy number variations; *TCP*, tumor cell percentage

#### Case 1

Initially, based on morphology, a dermal desmoplastic Spitz nevus was considered, but no *HRAS* mutation was detected. The lesion did not show cytomorphologic features of a DPN. Since multiple copy number variations (CNVs) were detected with SNP array (3 CNVs and chromothripsis of chromosome 22q), the lesion could not be classified as fully benign since presence of 3 or more CNVs is considered as indicative for malignancy [11]. However, morphology and immunohistochemistry did not clearly indicate malignancy. Therefore, the lesion was classified as an intermediate melanocytic tumor (MELTUMP—melanocytic tumor of unknown malignant potential—perhaps an AST). At the time of diagnosis of this lesion, no *MAP2K1* mutations in spitzoid lesions were reported in literature. The margins were free. A re-excision was performed showing no residual tumor.

#### Case 2

We considered this proliferation having spitzoid morphology, again with a *MAP2K1* mutation. In this case, there was loss of p16 staining. No features of a DPN were seen. Based on the six CNVs detected, it was decided to consider and treat the lesion as superficial spreading melanoma with spitzoid features (pTNM stage pT1a, TNM 8th Ed. 2017), and a re-excision with a margin of 1 cm was performed. In the re-excision specimen, no residual tumor was detected. No sentinel node procedure was conducted.

#### Case 3

This clinical atypical nevus (for clinical pictures, see Online Resource 4) showed spitzoid morphology with features of a Reed nevus. The single mitotic figure and focal ascent of melanocytes within the epidermis were considered a consequence of the encountered inflammation. With maintained expression of p16 and lack of CNVs, we had no suspicion of an intermediate or malignant lesion. Therefore, the lesion was classified as a variant Spitz nevus with features of pigmented spindle cell nevus of Reed with inflammatory changes and mild reactive atypia. Since the margins were free, no additional treatment was necessary.

#### Case 4

Clinically, the lesion was present for at least 4 years and unchanged (also based on repeated dermatological examination and unchanged clinical pictures, see Online Resource 4) without suspicion for malignancy. Also, based on cytomorphology, this melanocytic proliferation was regarded benign. A spindle cell nevus or desmoplastic Spitz nevus was considered. The lesion did not show classic DPN morphology. SNP array showed trisomy of chromosomes 5, 6, 11, 14, 15, and 20. Due to poor quality of the SNP array, presence of other CNVs could not be excluded completely. Gains of entire chromosomes in the absence of structural aberrations have been described as compatible with benign lesions in congenital nevi [12], outside the context of congenital nevi the meaning of numerical aberrations is unclear. Therefore, the lesion was classified as probably benign, at most low-grade MELTUMP (perhaps AST). A re-excision was performed showing no residual tumor.

## Discussion

We report four *MAP2K1*-mutated melanocytic tumors with different morphology ranging from benign to malignant but all sharing spitzoid morphological features. In about 20% of melanocytic neoplasms, including a proportion of spitzoid lesions, the genomic driver is thus far unknown. Despite the use of a broad panel of additional tests including NGS, immunohistochemistry, fluorescence in situ hybridization (FISH), or Archer techniques, no mutations, translocations, or amplifications can be demonstrated in a part of the spitzoid neoplasms. *MAP2K1* was added to the NGS panel in our practice on September 24, 2019. Since this date, 174 NGS analyses were performed on melanocytic lesions. The four reported cases were the first spitzoid neoplasms identified in our practice showing a pathogenic *MAP2K1* mutation, indicating a *MAP2K1* mutation is a rare genomic driver in these neoplasms. We consider the morphology of these lesions as clearly spitzoid and, in the absence of other specific mutations or translocations, we consider it highly likely that the *MAP2K1* mutation is the underlying genomic driver. We could not discern a clearly distinct phenotype within these *MAP2K1*-mutated lesions, although two cases resembled desmoplastic Spitz nevi. Furthermore, our four cases did not show a distinct phenotype compared with *MAP2K1* wild-type spitzoid lesions.

In the 4th edition of the 2018 WHO classification of skin tumors [13], lesions in the spitzoid pathway (pathway 4) are characterized by mutations in *HRAS*, tyrosine kinase fusions (*ALK*, *ROS1*, *RET*, *NTRK1/3*, and *MET*), or serine-threonine kinase fusions (*BRAF*, *MAP3K8*). It also mentions the presence of *MAP2K1* mutations in DPN lesions (pathway 1).

Up to now, *MAP2K1* alterations have hardly been reported in spitzoid melanocytic lesions, and *MAP2K1* alterations have not been reported in benign Spitz nevi yet. Last year, two spitzoid lesions with *MAP2K1* alterations were reported; one case of an AST [4] and one case of MST [9]. Our small case series confirms *MAP2K1* mutations can be present in lesions with a spitzoid morphology, and also shows the presence of this mutation in two (probably) benign cases.

Histologically, we saw relatively symmetrical melanocytic proliferations consisting of epithelioid to spindle-shaped cells. Cases 1, 2, and 4 demonstrated a comparable *MAP2K1* mutation resulting in the same deletion on protein level (p.Ile103_Lys104del). In case 1, desmoplastic stroma was seen and molecular diagnostics revealed 3 CNVs and chromothripsis of chromosome 22q, leading to classification as an intermediate lesion. Case 2 showed more cytonuclear atypia with immunohistochemical loss of p16; 6 CNVs were detected. Therefore, this lesion was classified as melanoma and treated as such. Case 4 did not show cytonuclear atypia or immunohistochemical signs of malignancy. SNP array revealed trisomy of 6 chromosomes without structural aberrations, based on which the lesion was classified as probably benign, intermediate at most. The same deletion (p.Ile103_Lys104del) was described by Quan et al. in an AST [4]. Amino acids 98 to 104 of MEK1 comprise an autoinhibitory domain, so a mutation in this region leads to activation of ERK that does not respond to feedback inhibition by RAS and RAF [4].

The third case showed no signs of malignancy by histology, immunohistochemistry, and additional molecular testing. It was classified as a variant Spitz nevus. This lesion showed a different activating *MAP2K1* mutation, namely a missense mutation: c.169A>G (p.Lys57Glu). This mutation has been described to disrupt the negative regulatory region of MEK1, resulting in increased ERK1/2 activation [14]. Although this specific mutation has not been reported in spitzoid lesions, it has been described in melanomas [14, 15].

Based on these four cases, we hypothesize that in a part of the spitzoid neoplasms, a mutation in *MAP2K1* is the initiating genomic event. *MAP2K1* mutations seem to be associated with intermediate or malignant spitzoid tumors both in our own cases and in a previously reported case [4]. This is also supported by the description of a case of spitzoid melanoma by Raghavan et al. [9]. They reported a *MAP2K1* mutation co-occurring with an *HRAS* mutation, mutations in *CDKN2A*, *ARID1A*, and *NOTCH2*, and a gain of chromosome 6p. Also, they described one melanoma case with an activating *MAP2K1* mutation with additional mutations in *PTEN*, *CDK4*, *ARID2*, *ATRX*, and *TP53* and a deletion of chromosome 6p. This case was not classified as a spitzoid melanoma, but based on our findings, this lesion could possibly also have a spitzoid signature. However, not all spitzoid lesions with a *MAP2K1* mutation show an intermediate or malignant phenotype since two of our cases with a mutation in this gene were classified as (probably) benign.

In conclusion, we describe four melanocytic lesions with spitzoid morphology harboring a *MAP2K1* mutation. Our small series and the few recently reported cases show that *MAP2K1* mutations can indicate a spitzoid genetic signature of a melanocytic lesion. In addition, we show that *MAP2K1* mutations can also be present in benign spitzoid lesions, but based on current limited data, *MAP2K1* mutations seem more frequently present in AST and MST. These data can possibly assist in further unraveling the molecular background of spitzoid neoplasms assisting in more objectively classifying these lesions and, eventually, better patient management preventing overtreatment or undertreatment.

## Electronic supplementary material

ESM 1 (PDF 167 kb)

ESM 2 (PDF 158 kb)

ESM 3 (PDF 374 kb)

ESM 4 (PDF 543 kb)
