# Supplementary material for: Expanding spectrum of “spitzoid” lesions: a small series of 4 cases with MAP2K1 mutations
Source: Virchows Arch. 2020 Oct 11;479(1):195–202. doi: 10.1007/s00428-020-02940-3 (PMC8298358; doi:10.1007/s00428-020-02940-3)
Supplement: Supplementary file 1 — (PDF 167 kb) [file 428_2020_2940_MOESM1_ESM.pdf]

Title

Expanding spectrum of 'spitzoid' lesions: a small series of 4 cases with *MAP2K1* mutations.

Journal

Virchows Archiv

Authors

K.G.P. Kerckhoffs[1], T. Aallali[2], C.A. Ambarus[3], V. Sigurdsson[4], A.M.L. Jansen[5], W.A.M. Blokx[5]

Affiliations

[1] Department of Pathology, Maastricht University Medical Center+, Maastricht, The Netherlands

[2] Pathology Expert Center, Hoorn/Zaandam, The Netherlands

[3] Department of Pathology, Sint Antonius Hospital, Nieuwegein, The Netherlands

[4] Department of Dermatology, University Medical Center Utrecht, Utrecht, The Netherlands

[5] Department of Pathology, Division of Laboratories, Pharmacy and Biomedical Genetics, University Medical Center Utrecht, Utrecht, The Netherlands

Corresponding author

K.G.P. Kerckhoffs

E-mail: kelly.kerckhoffs@mumc.nl

Online Resource 1: Details immunohistochemical stains

Most stains were performed or repeated at the University Medical Center Utrecht (UMCU). For Case 1, 2 and 3 few stains were performed at the center where the case was originally seen.

Immunohistochemical stains UMCU

**Stain**	**Clone**	**Firm**	**Dilution**
ALK	5A4	Novocastra	1:20
BAP1	C4	Santa Cruz	1:200
BRAF V600E	VE1	Ventana	RTU
HMB-45	HMB-45	DAKO	1:200
Ki-67	Ki-67	Ventana	RTU
MelanA	A103	DAKO	1:40
NTRK	EPR17341	Abcam	1:500
p16	MX007	Immunologic	1:800
p21	DCS-60.2	Cellmarque	RTU
ROS1	D4D6	Cell Signaling	1:50
S100	na	DAKO	1:8000

Additional immunohistochemical stains Symbiant Pathology Expert Center (Case 1)

**Stain**	**Clone**	**Firm**	**Dilution**
BAP1	BSB-109	BIO-SB	1:50

Additional immunohistochemical stains Meander Medical Center (Case 2)

**Stain**	**Clone**	**Firm**	**Dilution**
ALK	NCL-L-ALK	Novocastra	1:25
HMB-45	HMB-45	DAKO	RTU
MelanA	A103	DAKO	RTU
Ki-67	MIB-1	DAKO	RTU

Additional immunohistochemical stains Sint Antonius Hospital (Case 3)

**Stain**	**Clone**	**Firm**	**Dilution**
HMB-45	HMB-45	Ventana	RTU
MelanA	A103	Ventana	RTU
p16	E6H4	Ventana	RTU

na: not applicable; RTU: ready to use
