# Supplementary material for: Expanding spectrum of “spitzoid” lesions: a small series of 4 cases with MAP2K1 mutations
Source: Virchows Arch. 2020 Oct 11;479(1):195–202. doi: 10.1007/s00428-020-02940-3 (PMC8298358; doi:10.1007/s00428-020-02940-3)
Supplement: Supplementary file 2 — (PDF 158 kb) [file 428_2020_2940_MOESM2_ESM.pdf]

Title

Expanding spectrum of ‘spitzoid’ lesions: a small series of 4 cases with *MAP2K1* mutations.

Journal

Virchows Archiv

Authors

K.G.P. Kerckhoffs[1], T. Aallali[2], C.A. Ambarus[3], V. Sigurdsson[4], A.M.L. Jansen[5], W.A.M. Blokx[5]

Affiliations

[1] Department of Pathology, Maastricht University Medical Center+, Maastricht, The Netherlands
[2] Pathology Expert Center, Hoorn/Zaandam, The Netherlands
[3] Department of Pathology, Sint Antonius Hospital, Nieuwegein, The Netherlands
[4] Department of Dermatology, University Medical Center Utrecht, Utrecht, The Netherlands
[5] Department of Pathology, Division of Laboratories, Pharmacy and Biomedical Genetics, University Medical Center Utrecht, Utrecht, The Netherlands

Corresponding author

K.G.P. Kerckhoffs
E-mail: kelly.kerckhoffs@mumc.nl

Online Resource 2: Details Next-Generation sequencing and SNP array analysis

Amplicon-based targeted Next-Generation sequencing was performed in a diagnostic ISO-15189 accredited pathology laboratory as previously described [1] with an Ion Ampliseq™ custom designed panel (Cancer Hotspot Panel v2+3), including amplicons covering (parts of*) ABL1, AKT1, ALK, AMELY, APC, ARAF, ATM, BRAF, CALR, CCND1, CDH1, CDK4, CDKN2A, CSF1R, CTNNB1, DDX3Y, EGFR, ERBB2, ERBB4, EZH2, FBXW7, FGFR1, FGFR2, FGFR3, FLT3, GNA11, GNAQ, GNAS, HNF1A, HRAS, IDH1, IDH2, JAK2, JAK3, KDR, KIT, KRAS, MAP2K1, MDM2, MET, MLH1, MPL, MYD88, NOTCH1, NPM1, NRAS, PDGFRA, PIK3CA, POLD1, POLE, PTEN, PTPN11, RAF1, RB1, RET, ROS1, SMAD4, SMARCB1, SMO, SRC, STK11, TERT, TP53* and *VHL*. Briefly, library preparation was performed using the Ion Ampliseq™ Library kit 2.0 (Thermo Fisher Scientific, Waltham, USA) according to manufacturer's protocol on the Janus Express (PerkinElmer, Waltham, USA). Template preparation and chip loading was performed by the Ion Chef System using the Ion 510™ & Ion 520™ & Ion 530™ Chef kit and protocol. Sequencing was performed on the Ion Torrent S5, followed by variant calling by the Torrent Variant Caller (TVC). Variant annotation was done by an in-house bio-informatics pipeline using Ensembl API [1]. For *MAP2K1*, the NM_002755.3 reference sequence was used for annotation. Variants were visually inspected using the Integrative Genomics Viewer (IGV).

For SNP array analysis, first FFPE was restored using the Infinium FFPE DNA Restore Kit (Illumina, San Diego, USA). SNP array profiling was performed according to standard procedures using the Infinium CytoSNP-850k BeadChip (Illumina). SNP array data was visualized using NxClinical Software (BioDiscovery).

1. Strengman E, Barendrecht-Smouter FAS, de Voijs C, de Vree P, Nijman IJ, de Leng WWJ (2019) Amplicon-Based Targeted Next-Generation Sequencing of Formalin-Fixed, Paraffin-Embedded Tissue. Methods Mol Biol 1908:1-17. doi:10.1007/978-1-4939-9004-7_1
