# Supplementary material for: Expanding spectrum of “spitzoid” lesions: a small series of 4 cases with MAP2K1 mutations
Source: Virchows Arch. 2020 Oct 11;479(1):195–202. doi: 10.1007/s00428-020-02940-3 (PMC8298358; doi:10.1007/s00428-020-02940-3)
Supplement: Supplementary file 3 — (PDF 374 kb) [file 428_2020_2940_MOESM3_ESM.pdf]

Title

Expanding spectrum of 'spitzoid' lesions: a small series of 4 cases with *MAP2K1* mutations.

Journal

Virchows Archiv

Authors

K.G.P. Kerckhoffs[1], T. Aallali[2], C.A. Ambarus[3], V. Sigurdsson[4], A.M.L. Jansen[5], W.A.M. Blokx[5]

Affiliations

[1] Department of Pathology, Maastricht University Medical Center+, Maastricht, The Netherlands
[2] Pathology Expert Center, Hoorn/Zaandam, The Netherlands
[3] Department of Pathology, Sint Antonius Hospital, Nieuwegein, The Netherlands
[4] Department of Dermatology, University Medical Center Utrecht, Utrecht, The Netherlands
[5] Department of Pathology, Division of Laboratories, Pharmacy and Biomedical Genetics, University Medical Center Utrecht, Utrecht, The Netherlands

Corresponding author

K.G.P. Kerckhoffs
E-mail: kelly.kerckhoffs@mumc.nl

Online Resource 3

Microscopical and molecular findings case 1

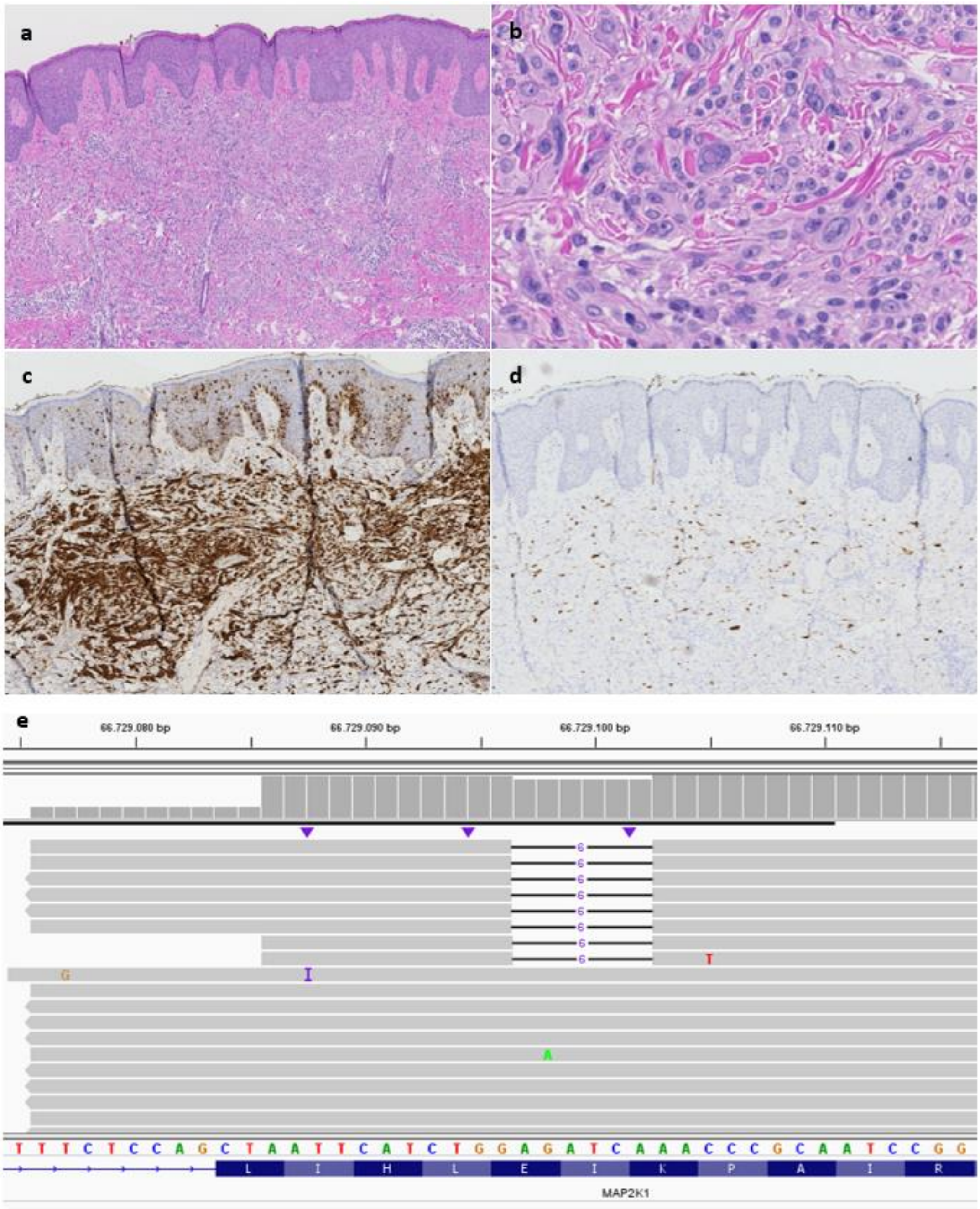

a H&E, x5: symmetrical, slightly wedge-shaped dermal melanocytic proliferation with overlying epidermal hyperplasia.

b H&E, x40: epithelioid to spindle-shaped melanocytes with pale, eosinophilic cytoplasm and in deeper sections more atypia with pleomorphism, prominent nucleoli and sometimes presence of nuclear inclusions.

c S100 stain, x5: positive staining.

d p16 stain, x5: few staining cells, but a checkerboard pattern could still be discerned.

e Integrative Genomics Viewer (IGV) visualization of the sequence data containing *MAP2K1* in-frame deletion c.306_311del (p.Ile103_Lys104del) in 13% of the reads (RefSeq NM_002755.3). The black lines indicate the location of the deletion.

Microscopical and molecular findings case 4

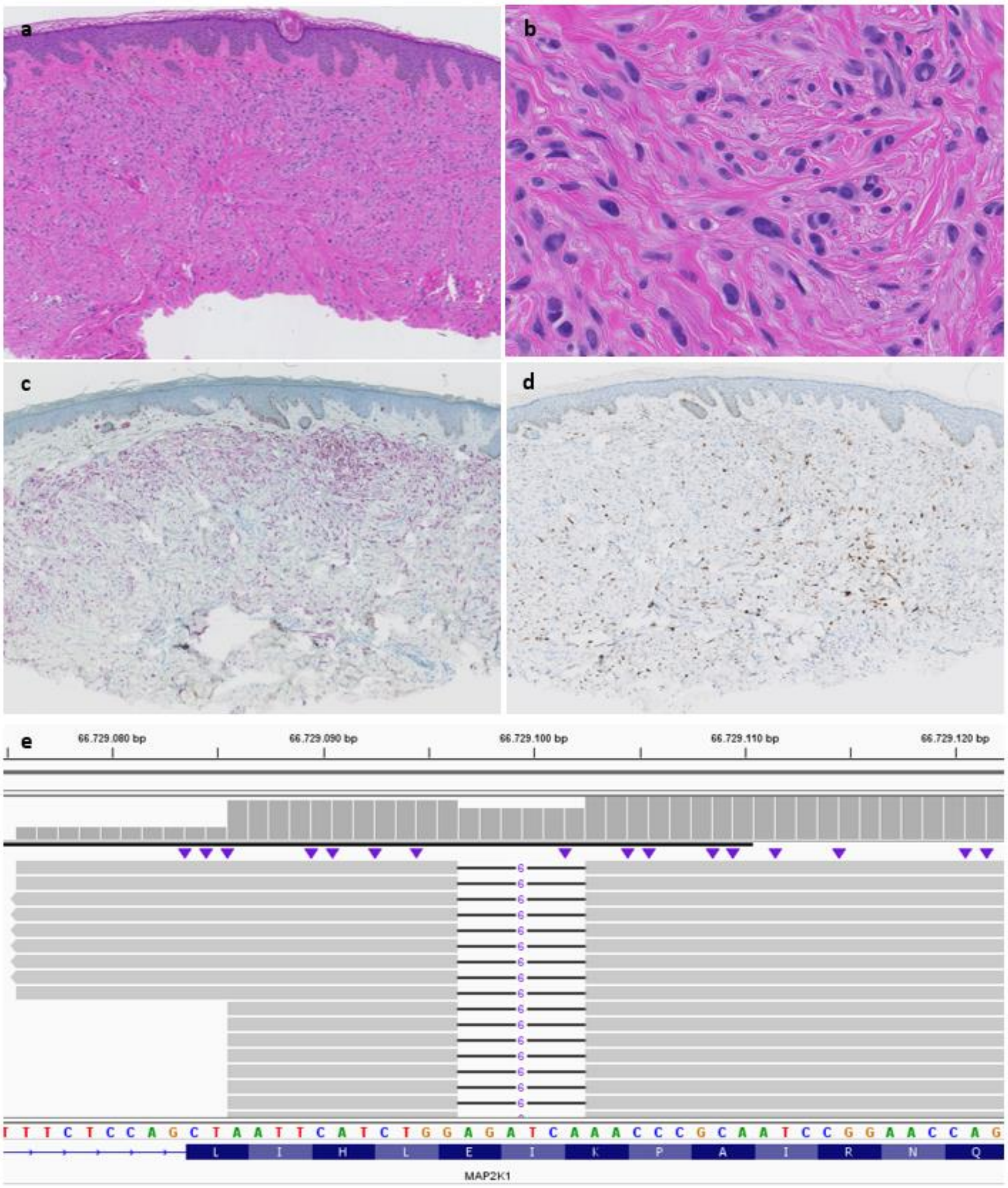

a H&E, x5: symmetrical, dermal, slightly wedge-shaped melanocytic proliferation showing maturation towards deeper parts.
b H&E, x40: spindle-shaped melanocytes with pale, eosinophilic cytoplasm.
c Melan A stain, x5: positive staining.
d p16 stain, x5: focal positivity.
e Integrative Genomics Viewer (IGV) visualization of the sequence data containing *MAP2K1* c.306_311del (p.Ile103_Lys104del) in 28% of the reads (RefSeq NM_002755.3). The black lines indicate the location of the deletion.
