# Supplementary material for: Expanding spectrum of “spitzoid” lesions: a small series of 4 cases with MAP2K1 mutations
Source: Virchows Arch. 2020 Oct 11;479(1):195–202. doi: 10.1007/s00428-020-02940-3 (PMC8298358; doi:10.1007/s00428-020-02940-3)

Title

Expanding spectrum of 'spitzoid' lesions: a small series of 4 cases with *MAP2K1* mutations.

Journal

Virchows Archiv

Authors

K.G.P. Kerckhoffs[1], T. Aallali[2], C.A. Ambarus[3], V. Sigurdsson[4], A.M.L. Jansen[5], W.A.M. Blokx[5]

Affiliations

[1] Department of Pathology, Maastricht University Medical Center+, Maastricht, The Netherlands
[2] Pathology Expert Center, Hoorn/Zaandam, The Netherlands
[3] Department of Pathology, Sint Antonius Hospital, Nieuwegein, The Netherlands
[4] Department of Dermatology, University Medical Center Utrecht, Utrecht, The Netherlands
[5] Department of Pathology, Division of Laboratories, Pharmacy and Biomedical Genetics, University Medical Center Utrecht, Utrecht, The Netherlands

Corresponding author

K.G.P. Kerckhoffs
E-mail: kelly.kerckhoffs@mumc.nl

Online Resource 4: Clinical pictures

Case 3

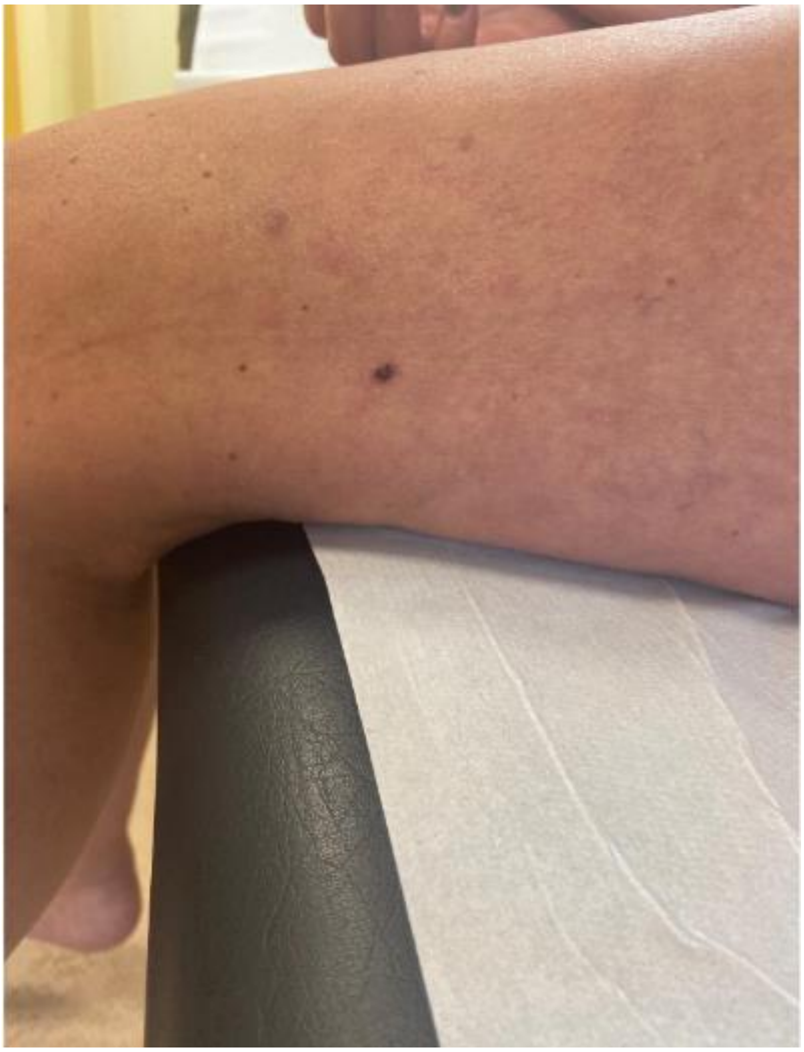

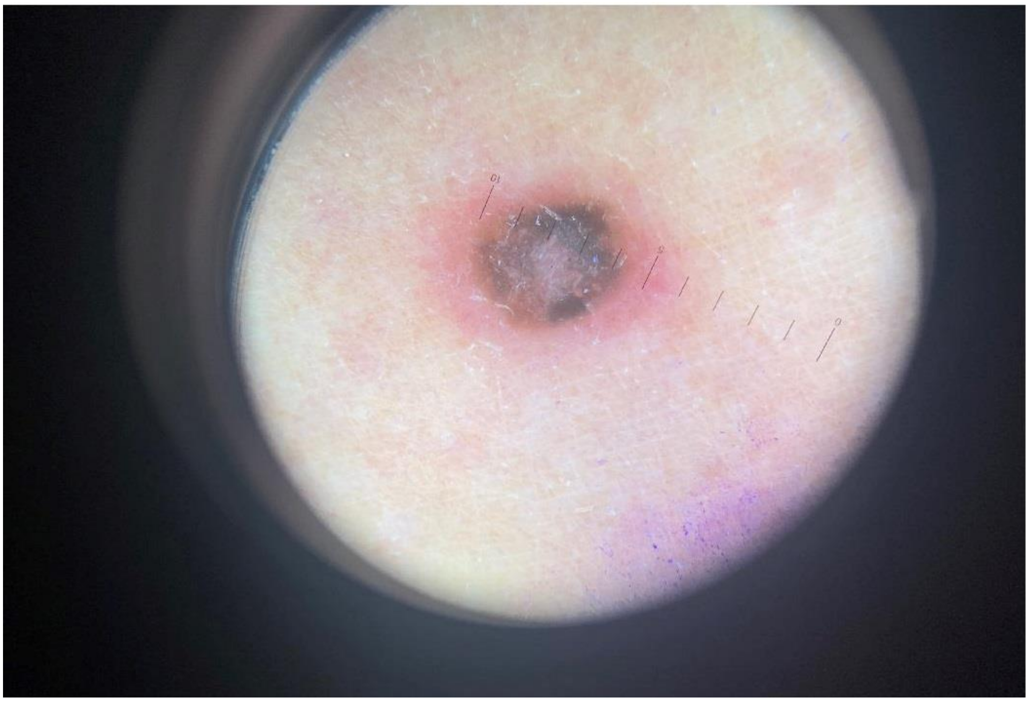

Case 4
In 2015

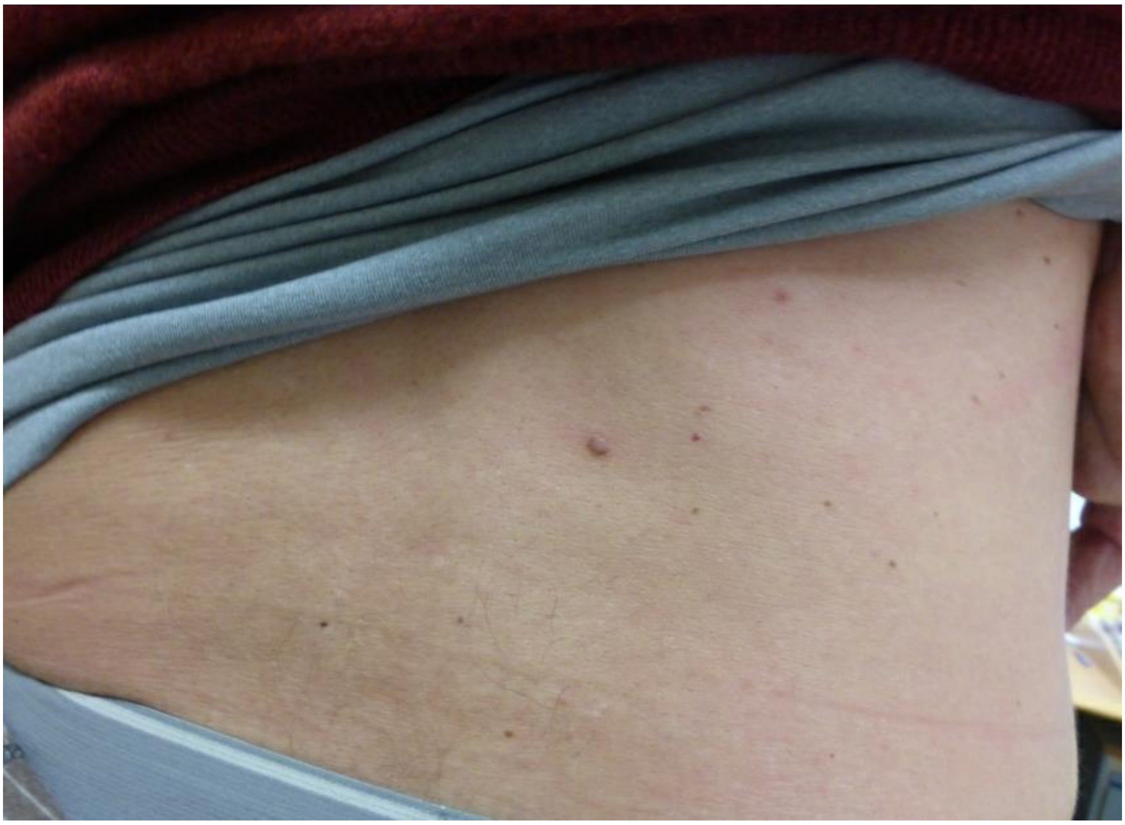

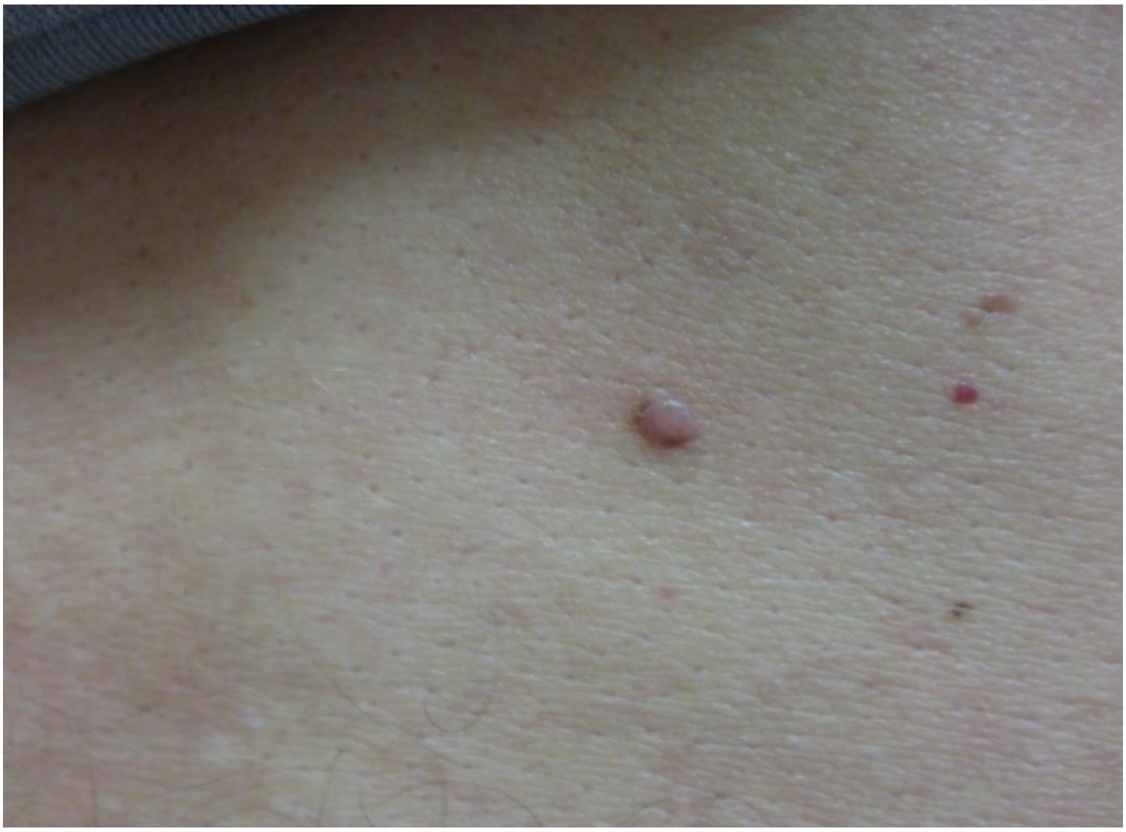

In 2019

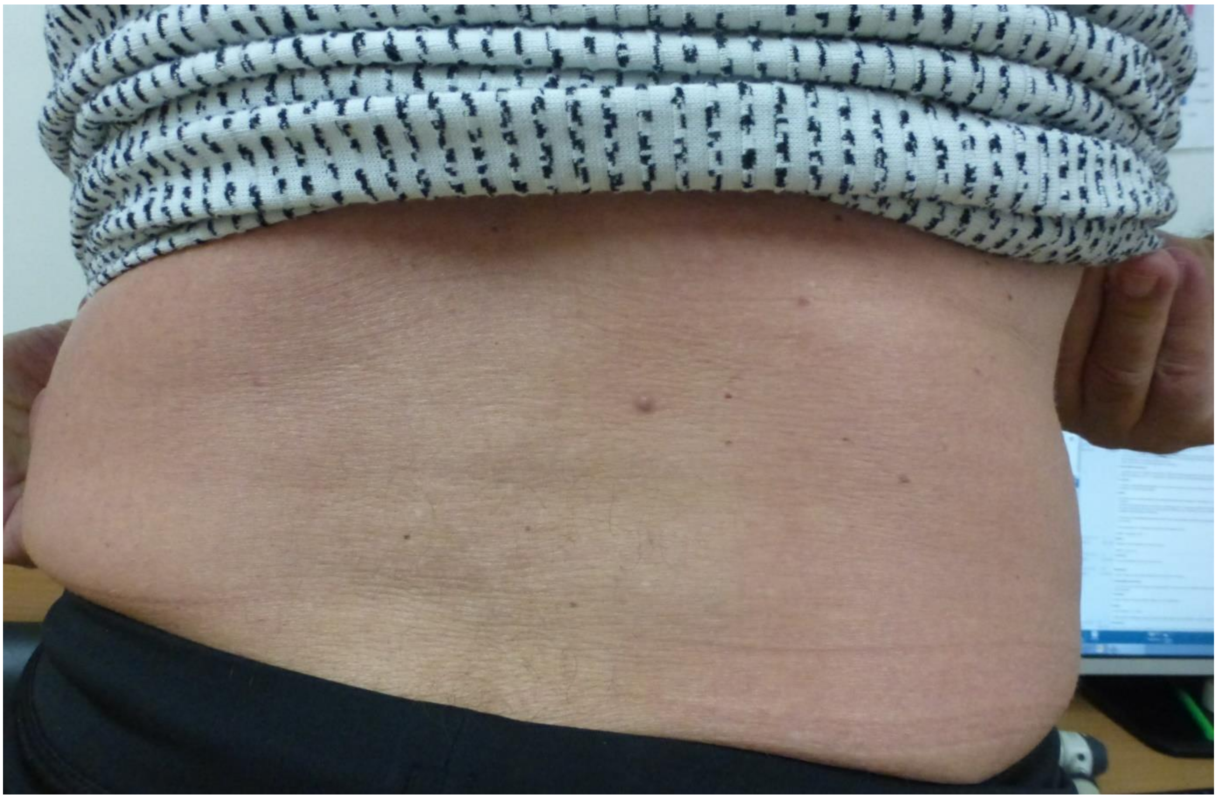

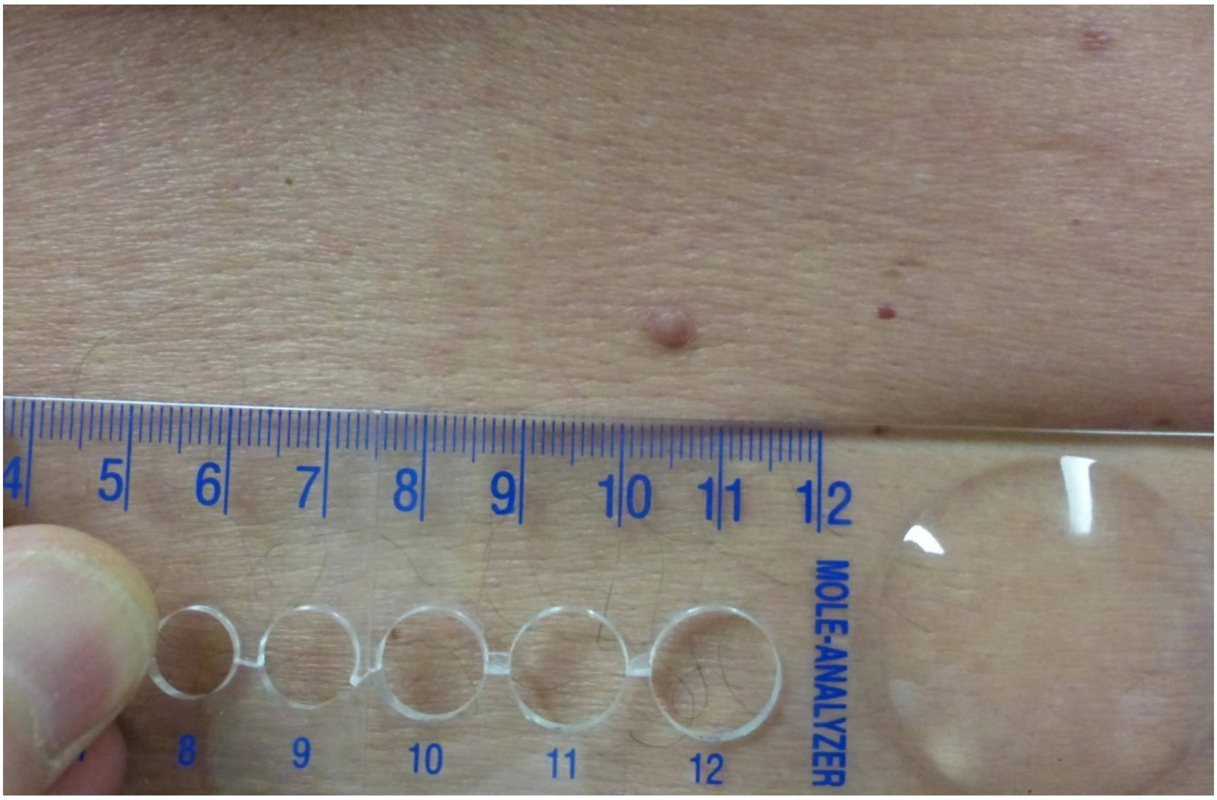

Supplement: Supplementary file 4 — (PDF 543 kb) [file 428_2020_2940_MOESM4_ESM.pdf]
